# Supplementary figures and images for: Bone-tissue decomposition of a single X-ray image via solving a Laplace equation
Source: PeerJ. 2025 Sep 11;13:e20016. doi: 10.7717/peerj.20016 (PMC12433621; doi:10.7717/peerj.20016)

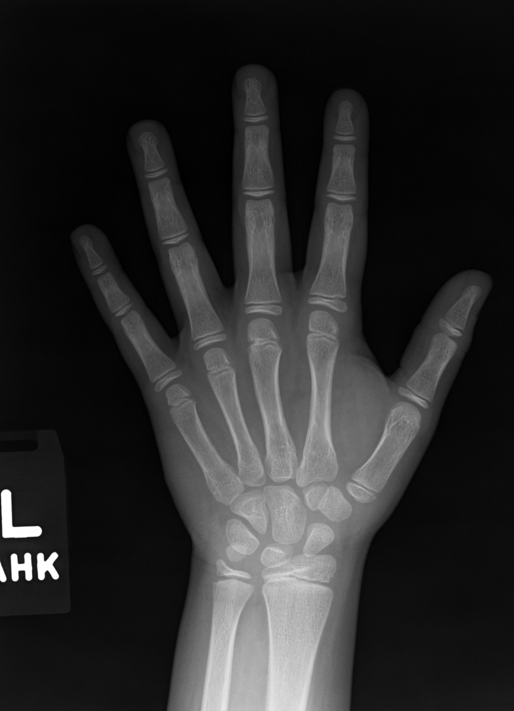

Supplement: Supplemental Information 1 — The matlab code shows the results from a simple example. [file peerj-13-20016-s001.zip › demo/input.png]

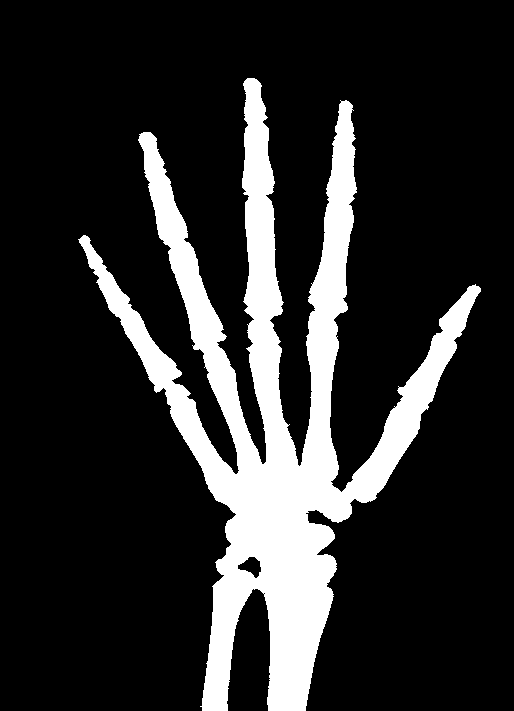

Supplement: Supplemental Information 1 — The matlab code shows the results from a simple example. [file peerj-13-20016-s001.zip › demo/mask.png]
